# An unusual presentation of atrial myxoma in an elderly patient: a case report

**DOI:** 10.1186/1757-1626-1-384

**Published:** 2008-12-10

**Authors:** Anneka Biswas, Ashish K Thakur

**Affiliations:** 1 Foundation year 2, Bradford Royal Infirmary, Duckworth Lane, Bradford, West Yorkshire, BD9 6RJ, UK; 2 Consultant Cardiologist, Dewsbury Hospital, Halifax Road, Dewsbury, WF13 4HS, UK

## Abstract

Left atrial myxoma is the most common intracardiac tumour. It could be seen in patients between 3–83 years of age, with the majority presenting in fifth decade of life as sporadic cases (90%) and second decade as familial cases (10%) [1]. It is an important source of central nervous system embolism [2]. Elderly patients often present with non specific symptoms that are often overlooked in the absence of a supporting cardiac history which makes an early diagnosis challenging. This case report discusses an unusual presentation of left atrial myxoma in an elderly patient.

## Case presentation

A 77 year old lady presented with a two week history of shortness of breath and acute onset palpitations and chest pain. She had also developed an acute confusional state for two days prior to her admission. She had been admitted five months ago with an episode of chest infection which was successfully treated with antibiotics. In the previous four years she had suffered from an episode of transient ischaemic attack (TIA).

Previous investigations had shown a normal computer tomography (CT) scan of the head, active rheumatoid arthritis and osteoarthritis. On this occasion she was afebrile, normotensive and had an irregularly irregular pulse rate of 150 bpm. Further cardiovascular, respiratory and abdominal examination was unremarkable and a detailed neurological examination did not reveal any significant abnormality.

**Figure 1:**
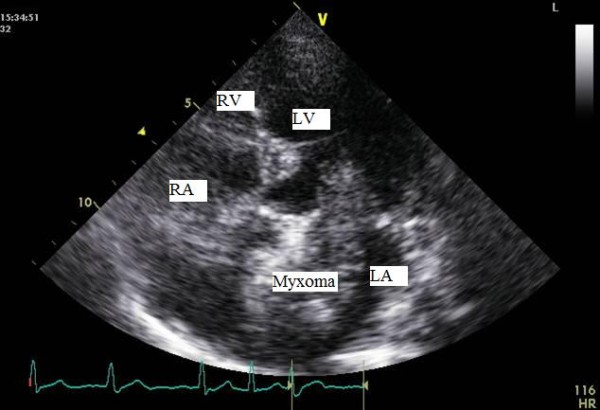
Transthoracic echocardiographic (TTE) image of the left atrial myxoma. The figure shows the tumor in the left atrium (LA) in the apical four chamber view, showing the four cardiac chambers (LA: left atrium, RA: right atrium, LV: left ventricle, RV: right ventricle).

**Figure 2:**
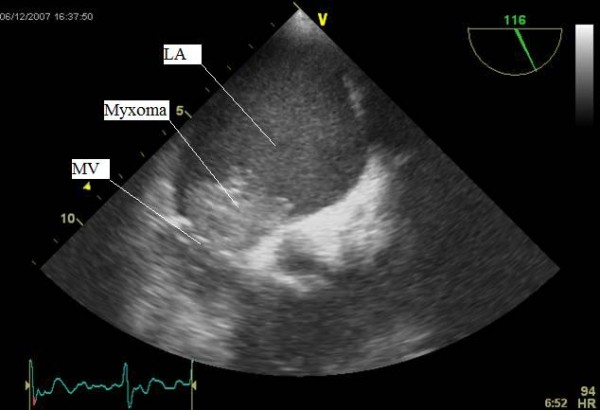
Trans-oesophageal echocardiographic (TOE) image of the left atrial myxoma attached to the atrial wall. The two chamber view shows the tumor attached to the atrial wall in the left atrium. Part of the tumor is also seen in the left ventricle due to diastolic prolapse (LA: left atrium, LV: left ventricle).

**Figure 3:**
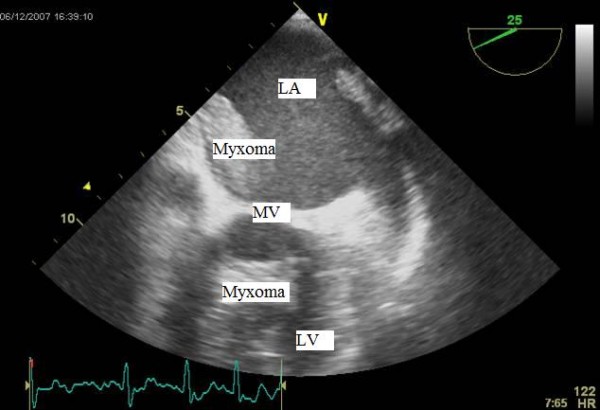
Trans-oesophageal echocardiographic (TOE) image of the left atrial myxoma obstructing the mitral valve. The two chamber view shows the tumor prolapsing through & obstructing the mitral valve in diastole (LA: left atrium, LV: left ventricle).

An electrocardiogram showed fast atrial fibrillation (AF) for which she was treated with digoxin, warfarin & beta blocker for further optimisation. An in-patient trans-thoracic echocardiogram (TTE) demonstrated a mobile mass in the left atrium (figure 1). Subsequent trans-oesophageal echocardiography (TOE) revealed a mobile large left atrial mass (2.7 cm × 3.5 cm) attached to the fossa ovalis region in the inter-atrial septum, prolapsing into the left ventricle through the mitral valve in diastole (figure 2 &3).

The patient declined surgical treatment in view of the risks involved, & died a few weeks later whilst on conservative therapy.

## Discussion

Atrial myxoma is the commonest (20–30% of all) primary intra-cardiac tumour in adults and two thirds of these arise in the left atrium [3]. Other locations are right atrium (next commonest), ventricles, superior vena cava or pulmonary veins. In 5 percent of cases myxomas can be multiple. Differential diagnosis [4] of atrial myxoma includes pedunculated thrombus, metastatic sarcoma and melanoma in left atrium. Other metastatic tumors could specifically metastasize via inferior vena cava to the right side of the heart & include hypernephroma, hepatoma, melanoma and intravenous leiomyomatosis from uterus.

Left atrial myxoma is most commonly seen in women with 90% being solitary and pedunculated and 10% being familial, with an autosomal dominant pattern of inheritance [5]. The mean age of onset is between 30–60 years.

Most myxomas produce symptoms when they weigh greater than seventy grams. The presentation of atrial myxoma can in three different ways:

• **Obstructive symptoms **– dysponea, cardiac failure, dizziness, collapse & syncope due to obstruction of the mitral valve.

• **Constitutional symptoms **– i.e. symptoms of autoimmune disease, vasculitis and various other non specific symptoms.

• **Embolic symptoms **– most frequently being cerebral emboli [6].

TOE has nearly 100% sensitivity for cardiac myxoma. Atrial myxoma is usually seen at the border of the fossa ovalis in the left atrium, attached to the inter-atrial septum as in this case. The tumor tissue manifests as spherical/pedunculated mass attached to the endocardial surface with hypoechoic areas [7]. TTE has less specificity than the TOE. Contrast CT demonstrates a well defined spherical or ovoid intracavitary mass. Magnetic resonance imaging (MRI) can visualise the point of attachment and helps differentiate a thrombus from a tumour. Differences in signal intensity between myocardium, tumor/thrombus is very helpful, especially with the use of contrast agent like Gadolinium-DTPA [4]. A cine MRI sequence is a very sensitive technique to distinguish between an thrombus and a tumor, intra-cardiac or intravascular. Surgical excision is the only definitive treatment for atrial myxoma. In relatively small tumors, TTE/TOE can be used to monitor the growth of the tumor, to decide the timing of the surgery [8]. Conservative management is of limited value in symptomatic patients with large myxomas. However, a conservative strategy with TTE/TOE monitoring, & anticoagulation is favoured in high operative risk patients, asymptomatic patients, and slow growing atrial myxomas.

The diagnosis of atrial myxoma can be elusive, especially when symptoms are suggestive of other diagnoses. In this case, the significance of this patient's past medical history of a transient ischemic attack only became apparent when the patient presented with new symptoms of AF, which led to various investigations looking for a source of cardiogenic cerebral embolism, eventually revealing the left atrial myxoma. This seems to be an unusual case due to the age at presentation of the patient. Left atrial myxoma presenting in seventh decade is rare, with only few published case reports, with this one of its first kind in the UK in the last ten years. Bire et al [9] studied the number of myxoma cases in patients over 75 years of age between 1962 and 1997 and found only 19 confirmed cases.

## Consent

Written informed consent was obtained from the deceased patient's next of kin for publication of this case report and accompanying images. A copy of the written consent is available for review by the Editor-in-Chief of this journal.

## Competing Interests

The authors declare that they have no competing interests.
